# Importance of cystic pedicle dissection in laparoscopic cholecystectomy in order to avoid the common bile duct injuries 

**Published:** 2016

**Authors:** E Suliman, RȘ Palade, E Suliman

**Affiliations:** * ”Carol Davila” University of Medicine and Pharmacy, Bucharest, Romania; ** First Surgery Clinic, University Emergency Hospital, Bucharest, Romania

**Keywords:** common bile duct iatrogenic injuries, laparoscopic cholecystectomy

## Abstract

Abstract

The dissection of the cystic pedicle represents the “main” issue in performing the cholecystectomy, as well as the surgical moment when many accidents may happen. The paper analyzes the most frequent causes, which can generate iatrogenic injuries of the common bile duct (CBD) during the dissection of the cystic pedicle, such as the ductal and vascular anatomical variants, the local pathological transformation, human errors, etc.

## Introduction

The iatrogenic injuries of the common bile duct (CBD) are rare but have a great postoperatory morbidity and mortality potential. This has generated many studies and complex analyses, which have shown that many of these serious intraoperatory accidents can be avoided. The surgeon is the person who has the entire responsibility of the surgery and he has to know very well the technical aspects of the surgical intervention, but he also has to have a thorough clinical and operatory experience in order to assure the total safety of the patient during laparoscopic cholecystectomy [**1**]. 

The “main” issue in performing the cholecystectomy is represented by the dissection of the cystic pedicle. It is not the only moment when CBD iatrogenic injuries can occur, but it is also the surgical moment when many accidents may happen. 

A thorough analysis of the causes that can generate CBD iatrogenic injuries during the dissection of the cystic pedicle, has shown that they are driven by the following: 

• ductal and vascular anatomical variants which hampers the correct identification of the structures [**2**] 

• pathological transformation or difficult surgical conditions, which modify topography and hamper the orientation and dissection [**3**] 

• human errors, when the surgeon has overestimated the limits of the surgery or the personal ones [**4**]. 

**Anatomical variants of the extrahepatic biliary ducts ** and the afferent vessels are more frequent than the “normal” descriptions presented in the classical anatomy treaties. Among the multitude of ductal and vascular variants, only about 10-15% have a technical surgical importance. 

The anatomoclinical studies have shown that these deviations from the normal anatomy, both of the vessels and of the biliary ducts are more frequent in patients with biliary lithiasis [**2**]. 

Due to the fact that the safety of the surgery depends on a correct anatomical and topographical orientation, the main ductal and vascular variants of the cystic pedicle, which are most frequently met in surgery, are presented. 

• The absent or very short cystic duct (5-10%) represents a congenital malformation or represents the result of a profound transformation due to an acute or chronic pathological process. Not being able to recognize this anatomical situation can generate CBD damage. 

• The double (duplicate) cystic duct is very rarely met. The second cystic duct drains in different topographic places: right hepatic duct, in a right hepatic accessory duct, at the level of the junction between the two lobar ducts (the origin of the common hepatic duct). 

• Multiple anatomical variants of the hepatocystic junction.

The specialty literature considers that in 80% of the patients it is important to find the classical description of the junction, in which the cystic duct opens on the right side of the common hepatic duct. This way, the most frequently used variants are described: 

− “Barrel shaped” cystic duct (7-20%)

− “Spiral shaped” cystic duct around CBD (5-35%) 

− Cystic duct opened in the right hepatic duct or in a right accessory hepatic duct (1-2%). This variant presents a major risk of injury of CBD intraoperatory. 

− Cystic duct opened in CBD on the posterior side, in the retropancreatic area or near Vater ampulla. 

From this data, the idea that for the safety of the surgery, not only the total dissection of the cystic duct and the compulsory identification of the hepatocystic junction in laparoscopic cholecystectomy should be followed, is highlighted [**2**]. 

**Accessory biliary ducts**, which open in the gallbladder.

They appear with a variable frequency (1-4%) and are known in the specialty literature as Luschka ducts. 

There are also superficial biliary ducts at the level of the cystic fossa, which may be injured during the gallbladder dissection, being responsible for some postoperatory biliary leakage.

**The vascular variants** are present in 50% of the cases. Not being able to recognize them can generate intra or postoperatory hemorrhages. The unexpected attempt of realizing the hemostasis can lead to CBD injury. 

The cystic artery originates (85%) in the right hepatic artery, situated posterior to CBD. It is divided into two branches, anterior and posterior, at different levels. During gallbladder dissection, important hemorrhages occur due to the injury of the posterior branch of the cystic artery, after the clipping of the anterior branch, considered the trunk of the artery. 

However, the cystic artery can have different origins: from the left hepatic artery, the common hepatic artery, the upper mesenteric artery, the gastroduodenal artery, cases in which, topographically, it does not have the classical way described at the level of Calot’s triangle anymore. Sometimes, the cystic artery has an important branch for the hepatic parenchyma. 

There are also accessory cystic arteries (20%), which can be found at the level of Calot’s triangle, but only sometimes (40-58%).

Other times, the cystic artery rapidly branches from the origin to a distance of 0,5 -1 cm. In these cases, the attempt of clipping the trunk can laterally crush the CBD or the right hepatic duct. In case of a vascular injury, the proximal end of the artery retracts and the hemorrhage is difficult to manage due to the short artery blunt [**5**]. 

In 5-15% of the cases, the right hepatic artery can appear in relation to the gallbladder infundibulum area. In these cases, it can be confused with the cystic artery. 

Moreover, in 13% of the cases there is a right accessory hepatic artery, which originates in the superior mesenteric artery, the common artery and sometimes even in the abdominal aorta. These cross the Calot’s triangle, being exposed to the danger of being injured during the dissection or being confused with the cystic artery. 

As a general rule, the dissection of Calot’s triangle must be done very carrefully, by clipping only the vessels that surely carry the blood to gallbladder (enter the gallbladder wall).

In 25% of the cases, there are also other vascular and/ or duct structures, beside the cystic duct and the cystic artery, at the level of the Calot’s triangle [**7**,**19**].

The CBD vasculature is provided by a vascular plexus, made up of vessels that originate in the pancreaticoduodenal arteries, the right hepatic artery, and the cystic artery. These make up two side arcades, median, and lateral, opposed to the CBD wall. The injury of these vessels can be followed by parietal necroses, sometimes stretched, and by late postoperative stenosis [**2**]. 

Difficult surgery conditions and/ or profound pathological transformations, which modify the topography and hamper the orientation and dissection [**6**]. 

There are many technical difficulties in laparoscopic cholecystectomy and limited ways of solving them, which makes the surgery not always be completed. 

The most frequent causes, which make the laparoscopic surgery difficult, are the following: 

**1.** Previous surgeries at the level of the abdomen, followed by a process of postoperative perivisceritis. Before performing a laparoscopic cholecystectomy, an adhesiolysis is required in order to reestablish the normal anatomy in the right subhepatic area. 

**2.** Obesity – in the obese people (3rd-4th degree), the subhepatic area is occupied by the transverse colon and the great epiploon, which make the correct exposure of the hepatic pedicle and the Calot’s triangle, difficult. 

**3. ** Difficulties in liver mobilization due to the great dimensions of the organ or the abnormal consistency of the parenchyma (steatosis, chronic hepatitis, cirrhosis). A massive square lobe or a Riedel lobulation, both create difficulties, which hamper the correct exposure of Calot’s triangle.

**4. ** The state of the walls and the gallbladder content can make the apprehensiveness and management of the organ, difficult. The evacuation puncture of gallbladder can improve the apprehensiveness and the easier topographic localization of the hepatic pedicle and the Calot’s triangle. 

**5. ** The infundibulocystic and pedicle lipomatosis hamper the correct identification of the structures at the level of Calot’s triangle. In these cases, it is advisable that the dissection is started on the right side (external), subserous at the level of gallbladder infundibulum, and, after the mobilization of this area, the dissection could be continued transversally, subserous, above the gallbladder wall, at a certain distance from the pedicle level. 

**6. ** Hartmann’s pouch, mostly when it is voluminous, adheres to CBD and hides the cystic duct, which is usually situated superiorly and posterior as compared to the gallbladder anterior side. The confusion of CBD with the cystic duct is usual, representing the most frequent error that generates an iatrogenic injury of the choledochus duct. 

**7. ** The short cystic duct or shorted by an inflammatory process creates major biliary stasis difficulties. The excessive traction on the infundibulum in order to obtain the lengthening of the cystic duct can lead to its disinsertion and the creation of a lateral CBD wound. This maneuver can place CBD in the form of an angle and, provided there is a lack of technical accuracy, the clipping can lead to the crushing of the choledochal wall and the appearance of late stenosis [**8**]

**8. ** Acute cholecystitis. The correct evaluation of the technical possibilities of solving the difficulties created by the acute inflammatory process is required. A wooden plastron, which is hardly dissectible, must determine the conversion to open surgery from the first moment. Due to the acute inflammatory process, the great volume of gallbladder, the satellite hepatic pediculitis, and the perivisceritis, the structures of Calot’s triangle are deeply modified [**9**]. 

Acute cholecystitis represents one of the most frequent causes of conversion to open surgery. Conversion is required when the anatomical identification is uncertain and the dissection is dangerous.

**9. ** Scleroatrophic cholecystitis creates major technical difficulties. The thick wall, molded on calculi, makes the apprehension of gallbladder difficult, its mobilization hard, and the cleavage plane during the dissection hard to find. Important transformations also happen at the level of Calot’s triangle, which make the identification of the structures hard. Dissection is hard, presupposes the loss of blood, and presents risks [**10**]. 

**10. ** The porcelain gallbladder represents a rarely met pathological entity, but which raises many technical problems, making the laparoscopic cholecystectomy difficult and risky [**11**]. 

**11. ** Hemorrhage represents a major conversion cause in laparoscopic cholecystectomy [12]. It appears in extremely different cases, but which must be well known in order to be prevented: 

• Large vessels at the level of the hepatic pedicle are rarely injured but can lead to the patient’s death. 

• Injuring the vessels that irrigate the gallbladder, especially the anatomical variants, which are not seen on time, can lead to difficult and dangerous cases. Bleeding can occur frequently from the cystic fossa, during gallbladder dissection, due to the posterior branch of the unclipped cystic artery. 

• By the locoregional inflammatory modifications, acute cholecystitis represents one of the most frequent causes of bleeding during cholecystectomy. 

• By embedding the vessels, the hepatic pedicle lipomatosis, creates hemostasis difficulties in the case of a careless and unexpected dissection. 

• Liver cirrhosis can lead to a continuous bleeding by many mechanisms. The portal hypertension syndrome, coagulation altering, presence of regeneration nodules at the level of the cystic fossa, are frequent causes of hemorrhage in laparoscopic cholecystectomy in liver cirrhosis [**13**]. 

The measures required to correctly realize hemostasis are very important: 

• A clear image is necessary, as well as a clean surgical field, unobstructed by blood and clots. 

• The irrigation-aspiration system must be handy and assure the correct cleaning of the surgical field. 

• Blind or excessive clipping and unexpected cauterization is forbidden without clearly identifying the bleeding source. 

• The vessel dissection, its identification, its clamping and correct clipping at sight is necessary. 

• After stopping the bleeding, surgical cholangiography is required in order to be sure that during the maneuvers no CBD injury has occurred. 

• Prevention means a careful hemostasis during the surgery, a correct dissection with the identification, preparation, clipping and sectioning of the vessels while being totally aware of the processes. 

• In order to avoid complications, in certain dangerous areas, supplementary precaution measures, attention, sagacious judgment, meticulous, gentle dissection, is needed. 

• If the control of hemostasis cannot be assured, mostly in the lack of a perfect anatomical orientation, conversion to open surgery is required. 

**Human error** occurs when the surgeon has overestimated the limits of the surgery or the personal ones. Human error can occur anytime during a surgical intervention [c]. These days, the surgeon is more and more subjected to a legislation, which requires the avoidance of all complications regarding the surgery [**15**]. However, some complications cannot be avoided (casual accidents, infections, bleeding, adverse reactions to applied therapy). 

Other complications, which are theoretically avoidable, are difficult to predict and control, usually being very complex [**16**]. 

In order to avoid the iatrogenic injuries of CBD in the case of laparoscopic cholecystectomy, a series of rules have been imposed in surgery, which must be well known and correctly applied: 

• In order to have a clear exposure of the subhepatic area, gallbladder fundus will be maximally lifted. 

• So as to correctly expose the Calot’s triangle, gallbladder infundibulum will be tracked to the left and inferiorly. This maneuver opens the angle between the cystic duct and CBD (raises the distance between the two ducts). 

• The most important landmark to identify the cystic duct is the infundibulocystic junction. Dissection must be started at the level of the vesicular infundibulum by the subserous mobilization of the right side of gallbladder. To continue, dissection will carefully progress from right to left. 

• The correct preparation of the cystic duct presupposes its circumferential dissection. This way, it will be completely detached from the other neighboring structures. Dissection starts from the identification of the infundibulocystic junction and it is realized near the gallbladder wall, after assuring that the duct is continued with the gallbladder [**17**]. 

• In order to acquire an optimal image, a telescope with a 30o opening, is preferred. This way we could control the entire circumference of the ducts and vessels [**18**]. 

• The area of Calot’s triangle must be carefully and meticulously prepared by removing the peritoneal folds, the adipose tissue, and the inflammatory bridges. Finally, the triangle area will be occupied by the cystic duct and the branches of the cystic artery, both structures continuing with gallbladder. Most of the times, we will be able to identify the right side of CBD, which assures the correct anatomical orientation (the visualization of the “triplet”). 

• The correct identification of the cystic duct will be followed by its safely clipping. The first clip must be applied at 0,5 -1 cm from the right side of CBD. The clip will be applied at sight, detached from the neighboring anatomical structures [**20**]. 

• In case a hemorrhage occurs, electrocautery or clips will only be applied at sight. This way, the major risk of injuring CBD is avoided [**12**]. 

• After the complete dissection of the cystic pedicle and its correct solving, the surgery will continue by strictly following the gallbladder wall, as far away from the CBD area and the hepatic hilum as possible. The use of cautery will be done at a certain distance from the clips placed on the cystic blunt or the cystic artery in order to avoid the spread of the current to the CBD wall (necroses, late stenosis). 

• In difficult cases, conversion to open surgery should not be avoided. This decision reflects professional maturity and does not represent a personal surgical failure [**8**]. „It is better to have an extra conversion than one conversion less” (Soper). 

## Conclusions

- Iatrogenic injuries of CBD represent the “Achile’s heel” in laparoscopic cholecystectomy. 

- Iatrogenic injuries of CBD more frequently occur in laparoscopic surgery as compared to open cholecystectomy. 

- In order to prevent their occurrence, a limitation of the laparoscopic technique is needed, strictly applied to the cases in which the safety of the surgery can be assured. 

- The surgeon must have a special training in laparoscopy technique, both theoretical and operatory, but also in open biliary surgery. 

- The surgeon must always have in mind “a plan of the game”, meaning a safe alternative for the anatomical orientation and the identification of the structures, a judicious maneuverability and skillfulness, always thinking that he has to avoid injuring the CBD, which can occur at the slightest inattention or uncontrolled gesture. 

**Acknowledgement:** This work received financial support through the project entitled “CERO – Career profile: Romanian Researcher”, grant number POSDRU/159/1.5/S/135760, cofinanced by the European Social Fund for Sectoral Operational Programme Human Resources Development 2007-2013.
